# Integrating perspectives in actinomycete research: an ActinoBase review of 2020-21

**DOI:** 10.1099/mic.0.001084

**Published:** 2021-09-13

**Authors:** Agustina Undabarrena, Camila F Pereira, Worarat Kruasuwan, Jonathan Parra, Nelly Sélem-Mojica, Kristiina Vind, Jana K. Schniete

**Affiliations:** ^1^​ Laboratorio de Microbiología Molecular y Biotecnología Ambiental, Departamento de Química & Centro de Biotecnología Daniel Alkalay Lowitt, Universidad Técnica Federico Santa María, Valparaíso 2340000, Chile; ^2^​ School of Pharmaceutical Sciences of Ribeirão Preto, University of São Paulo, Ribeirão Preto, Brazil; ^3^​ Microbial Cell Factory Research Team, National Center for Genetic Engineering and Biotechnology, National Science and Technology Development Agency, 113 Thailand Science Park, Khlong Nueng, Khlong Luang, Pathum Thani 12120, Thailand; ^4^​ Strathclyde Institute of Pharmacy and Biomedical Sciences, University of Strathclyde, Glasgow, G4 0RE, UK; ^5^​ Centro de Ciencias Matemáticas, Antigua Carretera a Pátzcuaro # 8701, Col. Ex Hacienda San José de la Huerta, Morelia C.P. 58089, Michoacán, México; ^6^​ NAICONS Srl, Viale Ortles 22/4, 20139 Milan (MI), Italy; ^7^​ Host-Microbe Interactomics Group, Wageningen University, De Elst 1 6708 WD, Wageningen, Netherlands; ^8^​ Biology, Edge Hill University, St Helens Road, Ormskirk, L39 4QP, UK

**Keywords:** ActinoBase, Actinobacteria, development, integrating perspectives, methodology, microbial ecology, natural products, regulation, specialised metabolites, *Streptomyces*

## Abstract

Last year ActinoBase, a Wiki-style initiative supported by the UK Microbiology Society, published a review highlighting the research of particular interest to the actinomycete community. Here, we present the second ActinoBase review showcasing selected reports published in 2020 and early 2021, integrating perspectives in the actinomycete field. Actinomycetes are well-known for their unsurpassed ability to produce specialised metabolites, of which many are used as therapeutic agents with antibacterial, antifungal, or immunosuppressive activities. Much research is carried out to understand the purpose of these metabolites in the environment, either within communities or in host interactions. Moreover, many efforts have been placed in developing computational tools to handle big data, simplify experimental design, and find new biosynthetic gene cluster prioritisation strategies. Alongside, synthetic biology has provided advances in tools to elucidate the biosynthesis of these metabolites. Additionally, there are still mysteries to be uncovered in understanding the fundamentals of filamentous actinomycetes' developmental cycle and regulation of their metabolism. This review focuses on research using integrative methodologies and approaches to understand the bigger picture of actinomycete biology, covering four research areas: *i*) technology and methodology; *ii*) specialised metabolites; *iii*) development and regulation; and *iv*) ecology and host interactions.

## Introduction

**Fig. 1.**
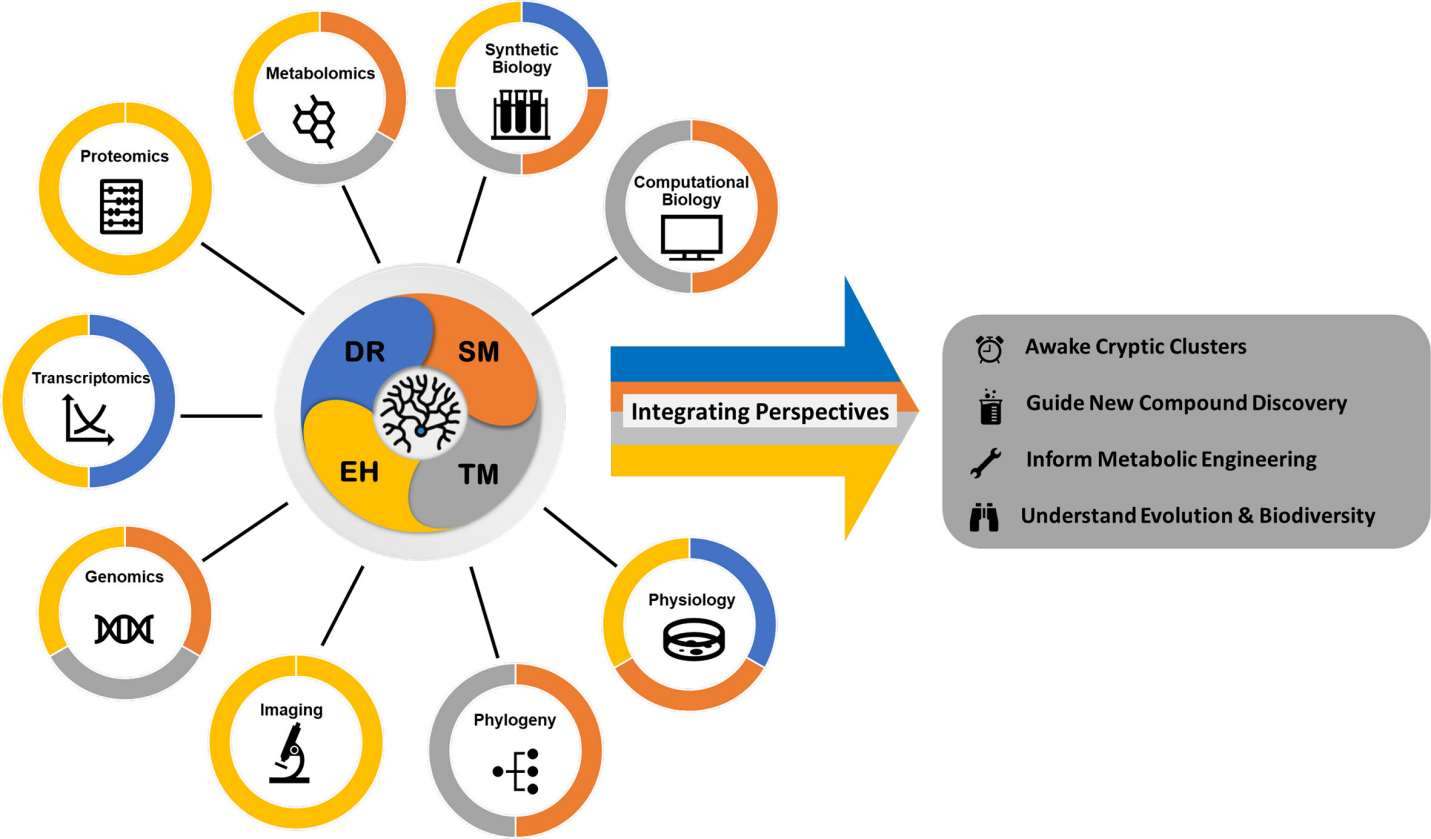
Integrating Perspectives in actinomycete research is accomplished by using a variety of different tools covering the four research areas: Development and Regulation (DR, blue); Specialised Metabolites (SM, orange); Technology and Methodology (TM, grey); and Ecology and Host Interactions (EH, yellow). The colour coding is based on selected publications covered in this review.

ActinoBase (https://actinobase.org) was established in 2019 with the support of the UK Microbiology Society as a Wiki-style initiative for the actinomycete research community. It aims to create a communal space to distribute knowledge and resources. Last year, ActinoBase published a review highlighting ten publications of important contributions to the field from 2019 [1]. This second review showcases 14 articles that integrate perspectives in actinomycete research from 2020 and early 2021, covering four main areas: *i*) technology and methodology; *ii*) specialised metabolites; *iii*) development and regulation; and *iv*) ecology and host interactions (Fig. 1).

Actinobacteria are Gram-positive high GC content bacteria, with a diverse range of morphologies ranging from unicellular cocci or rods to multicellular filamentous hyphae and spore-forming organisms depending on their genera [2]. They are widely distributed across ecosystems, ubiquitous in soil and water habitats, and can live in symbiosis with higher eukaryotes [3]. A striking feature of actinomycetes is that they produce chemically diverse specialised metabolites (SMs). SMs are compounds not essential for maintenance and growth, but often play a critical role in interspecies interactions [4]. Additionally, many SMs are of therapeutical value [5], constituting a rich resource for drug discovery [6]. SMs are often referred to as natural products (NPs) as well, this wider term encompasses any compound made by a living organism. Due to the current antimicrobial crisis, there is a need to improve the finding of novel antibiotics from actinomycetes, by developing advances in the detection/production pipeline, and applying integrative approaches to unveil SMs function [7]. For instance, SMs production in *Streptomyces* is linked to its life cycle [8–10], highlighting the importance of understanding its complex developmental regulation [11].

SMs are genetically encoded small molecules produced by a group of co-occurring genes referred to as biosynthetic gene clusters (BGCs), involved in synthesising one or more structurally-similar molecules [5, 12]. The genomic era has opened a new roadmap for the study of BGCs with genome mining as a prominent strategy to find candidates for novel bioactive compounds [4, 13, 14]. This has allowed researchers to uncover thousands of new BGCs of different classes, including polyketides (PKs); non-ribosomal peptides (NRPs) [15–18]; PK-NRP hybrid compounds; ribosomally synthesized and post-translationally modified peptides (RiPPs) such as lanthipeptides or thiopeptides [19–21]; and volatile organic compounds (VOCs) [22, 23]; among others [24–28].

Although genomics has revealed key elements of SMs biosynthesis, there are still challenges to overcome. Their low production, the rediscovery of known compounds, and a general lack of understanding of the role of these compounds in nature, as well as the fact that many organisms remain unculturable [29, 30]. Moreover, many computational tools for high-throughput data mining have been developed to support BGCs prioritisation and dereplication [31]. Handling big data requires prioritisation to establish selection criteria for certain BGCs based on the specific research context [32]. Dereplication is selecting a BGC based on its novelty, often achieved by comparing with databases such as Minimum Information about a Biosynthetic Gene Cluster (MIBiG) [33, 34].

Additionally, advances in synthetic biology have improved the genetic manipulation of actinomycetes, helping to characterize and functionally validate BGCs predictions [35–37]. Furthermore, engineering BGCs has been crucial to unlocking their potential since most BGCs are silent or cryptic under controlled laboratory conditions [38]. Recent definitions for cryptic and silent BGCs describe cryptic as a BGC where the compound is unknown, but its biosynthesis is not; and silent as the compound is undetectable [38]. In this context, understanding actinomycete relationships with the environment and other organisms is a crucial factor when searching for BGC elicitors, referred to as molecules or signals that can trigger the expression of a certain BGC [11].

Currently, integrative approaches are used to fill these knowledge gaps, which ultimately deepen our understanding of actinomycete biology. This trend is the central theme of this review, which highlights recent studies from 2020 and early 2021 that integrated genomic, metabolomic, transcriptomic, genetic engineering, and ecological data to provide significant insights in actinomycete research. (Fig. 1).

## Technology and methodology

Stagnation in the traditional SMs discovery pipeline has motivated researchers to develop novel strategies for compound discovery [7]. The boost in sequencing has guided genome-based predictions of BGCs with tools such as antiSMASH for their classification, according to their core enzymes [39]. Similarly, PRISM [40], BAGEL [41] or RiPPMiner [42] are complementary mining tools, and a complete list is available here http://wwwsecondarymetabolitesorg/mining/. Development of community resources such as MIBiG repository have been essential for deposit functionally characterised BGCs data in a standardised manner [34]. A continuous challenge is to interpret BGCs in terms of their novelty and function, for which tools facilitating their comparison applied to large data sets have invigorated clinically relevant SMs discovery. Two of these recent tools, BiG-SCAPE [43] and Global Natural Product Social Molecular Networking (GNPS) [44], enable building of interactive similarity networks of BGCs and SMs, respectively. While grouping of BGCs is according to sequence similarity, the grouping of SMs is by mass spectrometry fragmentation patterns (MS/MS). In the past year, these tools have been expanded by the release of BiG-SLiCE [8], BiG-FAM [45] and Qemistree [46], allowing further refinement of big data exploration, which we have selected to address in detail below. A comprehensive general review of the latest bioinformatics development is also offered in Medema (2021) [47].

In parallel, the field has encountered important methodological advances in synthetic biology. This complementary approach is simplifying the experimental testing of the hypotheses derived from computational predictions. Actinomycetes are often hard to genetically manipulate: some exhibit low intrinsic recombination frequencies, are slow-growers, or fail to sporulate; thus, many efforts go to delivering or adapting new tools for their genetic manipulation [48]. Traditionally PCR based technologies such as ReDIRECT [49] have been complemented or replaced by other methods such as the meganuclease I-SceI from *Saccharomyces cerevisiae* [50] for the use in novel and genetically uncharacterised actinomycetes; the adaptation of the CRISPR-Cas9 system through the development of pCRISPomyces [36]; and more recently to the CRISPR-BEST system [51], which allows genome manipulation without the introduction of a double-strand break, highlighted in last year’s ActinoBase review [1].

Another challenge in metabolic engineering is that engineered strains frequently become less fit due to unforeseen metabolic constraints and imbalances, necessitating individual and optimisation for each strain and heterologous cloned BGCs. Thus, the characterisation of new biosynthetic pathways becomes a very time consuming and laborious process when testing expression in different heterologous hosts. A detailed review focussing on synthetic biology advances in *Streptomyces* can be found at Breitling *et al*. [37].

To have a toolbox of entirely synthetic systems controllable for each subclass of BGCs would allow a more streamlined screening of the large amount of cryptic and silent gene clusters that we observe in the *in silico* analyses [52]. We have chosen to highlight two articles with important advances in these topics: an optimised cell-free transcription-translation one-pot reaction that circumvents heterologous hosts' incompatibility and bring us a step closer to the above-mentioned synthetic biology toolbox [53]; and the combination of a quorum-sensing mechanism inside a CRISPRi circuit, allowing a dynamic metabolic engineering approach [54].

### BiG-SLiCE: A highly scalable tool maps the diversity of 1.2 million biosynthetic gene clusters [8]

One approach to understanding the vast diversity and taxonomic distribution of BGCs is through their comparative study. Homologous BGCs are hypothesized to produce similar SMs, which can be grouped based on sequence similarity into gene cluster families (GCFs). The first tool to accomplish this was BiG-SCAPE which allowed calculation of pairwise distances between BGCs and mapped them onto interactive sequence similarity networks [43]. However, the amount of computing power required by this algorithm was a drawback to scale up from thousands to millions of BGCs. To address this problem, BiG-SLiCE was developed [8]. In contrast to the detailed BiG-SCAPE classification, BiG-SLiCE manages big data by representing BGCs in the Euclidean space, grouping them in non-linear and non-pairwise fashion. In this way, this powerful clustering platform manages to roughly organise 1,225, 071 BGCs from genomes and metagenome-assembled genomes (MAGs) in just ten days.

BiG-SLiCE operates by finding a set of 121, 299 BGCs as centres of GCFs after the first round of clustering, assigning BGCs to its corresponding family by considering the minimal distance to these centroids. For centroids determination, BiG-SLiCE firstly encodes BGCs into a numerical vector capturing the presence of selected domains from a curated set of Hidden Markov Models (HMM) from the Pfam database [53]. Subsequently, the BIRCH superlinear clustering algorithm is applied to these numerical vectors to define family profiles [55], and the average vector of the cluster will be determined as the centroid. Centroids are then directed to the second round of clustering, using the K-mean algorithm to allow visualisation in a species tree [56]. As a result, families are grouped into 500 bigger sets called bins. Afterwards, every BGC is mapped to the closest centroid of these bins and are further classified. Finally, SQLite is used to store this data and allow new BGCs searches (https://www.sqlite.org/index.html), while R and python scripts are provided as visualisation tools.

For the first time, a global map of biosynthetic diversity was created thanks to BiG-SLiCE: 121, 299 centroids from the first round of clustering were grouped into 500 bins, and finally, 1.2 million BGCs were mapped onto these. The 500 bins are displayed in a hierarchical tree allowing exploration of their distribution across different taxa. While *Streptomyces* confirms its status among the genera with the greatest BGCs potential, other members of *Actinobacteria* and Ascomycota, Firmicutes, and Proteobacteria phyla also appear among those bearing important biosynthetic potential. BiG-SLiCE also estimates that only 3.7 % of the GCFs are closely related to the characterised MIBiG BGCs, highlighting the unknown chemistry that remains uncovered. Another advantage of BiG-SLiCE is that it allows to rapidly locate a new BGC of interest via a simple search mode in the pre-calculated families stored in the BiG-FAM database.

Although BiG-SLiCE was developed to reveal the uncharted biosynthetic potential, it is based solely on antiSMASH-predicted BGCs, and a selection of biosynthetic Pfam domains was used for the construction of the GCFs models. Consequently, genomic vicinities predicted by other algorithms, or BGCs lacking the specifically selected domains, would be left out of the analysis. Hopefully, this will improve in the future. Moreover, due to gene content or sequence variation, each GCF may be subdivided into subsets of BGCs that produce different metabolites. The number of different products by family may differ as well as the genetic variation rate. A remaining challenge is to incorporate different thresholds to integrate each family, considering the chemical variation of its produced metabolites.

### BiG-FAM: the biosynthetic gene cluster families database [45]

The increasing availability of BGCs data created the problem of understanding their diversity on a new computational scale. Specific BGCs databases, such as antiSMASH-DB [57] and IMG-ABC [58], can display similarity to the MIBiG repository. However, they do not consider a metric of distance to homologous BGCs conforming GCFs. Thus, there was a lack of a BGCs family database, now fulfilled by the development of BiG-FAM [45]. While BiG-SLiCE solved the problem of defining a metric and algorithm in feasible computational time that allow the classification of the millions of currently available BGCs into GCFs, the BiG-FAM database acts as a repository to display BiG-SLiCE results.

In BiG-FAM, SQL is used to provide fast searches (i.e. 0.5 s) from BiG-SLiCE pre-calculated families, while Python/R scripts and flask libraries (Python-based web framework) provide a user-friendly interface. BiG-FAM goes beyond a storage and consulting website, as it allows exploring taxonomic distributions by including a web-based query function for users to match their own antiSMASH predicted BGCs onto GCFs, which reveals the closest and distant relatives of the queried BGCs. Meaning that a BGC of interest, not included in the BiG-SLiCE calculation, could easily be situated in BiG-FAM families without the necessity of calculating the distance between the BGC of interest and every other BGC represented in BiG-FAM. Subsequently, BiG-SLiCE can calculate the closest centroid and finally, classify the BGC of interest into the corresponding family. Another advantage of BiG-FAM is that it includes functionally characterised BGCs from MIBiG, accessible by either using a taxonomy search or other filters of interest. The BiG-FAM result is displayed as an interactive platform by providing graphic visualisation of Pfam domains within each gene and creating a word cloud to show the abundance of these domains.

Authors showcase BiG-FAM with two examples. First, a ranthipeptide (i.e. radical non-α thioether containing peptides) RiPP BGCs diversity was explored, where ranthipeptide genetic neighbourhood and the precursors needed for its biosynthesis were discovered using a comparative visualisation. Secondly, BGCs assignment into GCFs from a newly sequenced *Streptomyces* strain was carried out, and the novelty of ‘Region 15.1’ BGC was explored by localizing its family. Using ClusterBlast, this BGC showed low levels of similarity against public databases, consistent with BiG-FAM results, which showed it as a singleton.

BiG-FAM allows similarity searches by adding of another layer of comparison, such as domain architecture, which can add knowledge into more distant BGCs relationships. It can explore homologous BGCs as families and furthermore, it can place BGC of interest within these families. BiG-FAM opens the door to address questions regarding the composition and variation within these families, and their analysis regarding conservation. Nevertheless, as BiG-FAM is not a BGC database, it does not provide BGC specific details, only family information. Pre-calculation and previous knowledge are required to define parameters such as the minimum set of domains that allow vectorization of BiG-SLiCE, and it can be a drawback when analysing underrepresented clades, such as Archaea, which genomes are one order of magnitude below other domains. Thus, results might be biased towards overrepresented groups.

An important issue that remains for future work is to understand how to apply these algorithms to other biological processes involving gene clusters, such as operons from central metabolism or unknown BGC classes. The ability to classify and map processes other than specialised metabolism in this way would increase our understanding of specialised metabolite producers. Additionally, metadata such as geographic or ecological distribution of the BGCs families could be incorporated into the database, as it could help answer evolutionary, genomic, and ecological questions regarding BGCs.

### Chemically informed analyses of metabolomics mass spectrometry data with Qemistree [46]

While BiG-SLiCE and BiG-FAM rely on genomic input, metabolomics has also encountered the necessity to develop tools to access big data to extract meaningful information. For instance, GNPS molecular networking [44] allows MS/MS comparison to visualise their relations in discrete molecular families (MFs). In contrast to classical molecular networking, Qemistree enables exploring the relatedness of all features in the dataset. Qemistree is a tree-based metabolomics visualisation tool for tandem MS data from untargeted metabolomics experiments, hierarchically representing chemical features, analogous to phylogenetic tree building for sequences.

The pipeline utilises chemical feature detection [59] followed by SIRIUS [60, 61] to determine each feature’s molecular formula, and using the fragmentation pattern to estimate the best fragmentation tree. In the next step, CSI: FingerID [62] uses those fragmentation trees and kernel support vector machines to predict molecular properties resulting in molecular fingerprints. Pairwise distances are then calculated and hierarchically clustered to generate a tree representing structural relationships. Finally, ClassyFire [63] can assign chemical taxonomy and iTOL acts as a visualisation tool for the resulting tree [64]. The software pipeline is freely available either as a QIIME2 plugin [65] or GNPS networking flow [66].

The authors utilise three different approaches to evaluate the tool by case studies. The first one attempts to demonstrate Qemistree’s advantage over traditional analysis tools relying on molecular fingerprints, independent of retention time shifts that could result from differing elution conditions. The second one shows that the tree-based analysis using chemical relatedness can detect similarities among different samples, where traditional methods often lead to an over-assumption of differences. Last, a heterogeneous dataset was obtained, showing that the chemical features agreed with ClassyFire, confirming that molecular fingerprints are a suitable marker.

Qemistree relies on molecular fingerprints and their prediction is limited to the quality and coverage available in MS/MS spectral databases. In the future, it will be interesting if the concept of Qemistree can be applied on other chemical relationship markers such as chemical classes, spectral motifs and shared biosynthetic origins, however this will require further benchmarking to assess when to use which approach.

Overall, Qemistree is universally applicable and a readily available tool to interpret and visualize complex metabolomics data. As GNPS is already widely used in the actinomycete community, this additional feature in the pipeline will be of value to compare samples from different taxa and/or environments to discover novel chemical diversity and aid dereplication challenges at a large scale, like the Earth Microbiome project [67]. Moreover, it adds another layer of understanding, taking into consideration molecular relatedness in addition to the genetic one.

### A *Streptomyces venezuelae* cell-free toolkit for synthetic biology [68]

Cell-free systems are powerful tools in synthetic biology, allowing a controlled and simplified environment to study specific cellular processes in the absence of the entirety and complexities of cells [69]. Due to mostly unknown regulatory circuits for silent BGCs, cell-free systems stand as an attractive and fast wet-lab option to test hypotheses derived from computational predictions.

Moore *et al*. introduced an optimised cell-free transcription-translation (TX-TL) system tailored for studying *Streptomyces* silent metabolic pathways. Cell-free TX-TL systems are crude cell extracts (or purified ribosomes) containing translation factors and the respective DNA coding for the pathway of interest on a plasmid in a buffered solution. Thus, it allows a quick and inexpensive way of studying transcription, translation and biosynthesis in a ‘one-pot’ reaction [70–72]. It offers a simplified and controllable environment to study SMs by providing precursors for biosynthesis and direct control over feeding precursors in short experimental time scales. Moreover, it is crucial for circumventing unknown regulatory cascades by providing the genes of interest on a plasmid under the control of a chosen promoter.

A robust, high-yield *Streptomyces* TX-TL protocol is achieved by optimising temperature; pH; regulatory elements (i.e. promoter strength, start codon preference, terminator); energy solution (i.e. NTP concentration); ATP regeneration (i.e. secondary energy sources); RNase inhibition (i.e. polyvinyl sulfonic acid (PVSA) concentration); DNA concentration and methylation, yielding at least six-fold improvement to the previous system with fewer batch variations.

Protein expression yields were assessed by codon optimised expression of genes for sfGFP, mVenus and mScarlet as reporters using real-time detection, as well as the expression of the oxytetracyline enzymes (*otc* cluster) from *S. rimosus *, and three NRPS genes (*txtA and txtB* from *S. scabiei *; and an unknown NRPS from *S. rimosus *). Furthermore, they also studied the expression using a codon optimised GUS reporter and two pathways from *S. venezuelae* DSM-40230: the copper metallochaperone (MelC1) and tyrosinase (TyrC) from the melanin pathway, as well as the early-stage *haem* pathway (HemB, HemD-CysG, HemC). In both cases, the TX-TL systems supported transcription, translation, and biosynthesis, as they observed the brown pigment for melanin by UV light absorption and intermediate products from the *haem* pathway by LC-MS analysis.

Overall, this work is a promising proof of concept for using cell-free extracts in SMs research, which may lead to the characterisation of unknown BGCs from genome mining. However, further challenges remain in applying it to environmental strains and other classes of specialised metabolites, where less *a priori* knowledge is available. Nevertheless, this tool has potential for scalability that could lead to the chemical purification and characterisation of new metabolites, circumventing incompatibilities with heterologous hosts, a typical obstacle in traditional approaches [73].

### Developing an endogenous quorum-sensing based CRISPRi circuit for autonomous and tuneable dynamic regulation of multiple targets in *Streptomyces* [54]

Traditional metabolic engineering often referred to as static metabolic engineering, mainly uses two approaches to increase metabolite titres: either by deleting genes from competing pathways to force the metabolic flux into a specific pathway, or by overexpressing key enzymes through engineering promoters or ribosome binding sites [74]. However, this often results in an overall reduced strain fitness caused by metabolic imbalances due to competition around cellular resources, leading to the accumulation of certain undesired products and/or decreased growth rates. The formation of heterogenous microenvironments inside large scale bioreactors can also lead to variable performance of statically engineered strains. A comprehensive overview of the challenges can be found in Hartline *et al*. [75].

Dynamic metabolic engineering approaches attempt to create an autonomous response based on the environmental factors or internal metabolic states. Tian *et al*. introduce a pathway-independent regulation using a CRISPR-based tool with integrated quorum sensing (EQCi) to regulate multiple target genes in *Streptomyces *. The quorum sensing component allows an autonomic and dynamic response by the strain to its environment [76]. The authors designed an EQCi circuit to respond in a density-dependent manner using a γ-butyrolactone (GBL) responsive promoter to drive dCas9 expression and a synthetic promoter to regulate the transcription of guide RNAs (gRNAs) targeting genes of interest. GBLs are well-known for playing a role as regulatory molecules for antibiotic production and development in *Streptomyces* [77] which counteract the aforementioned challenges.

The system was validated with rapamycin, a polyketide produced by *S. rapamycinicus *, by perturbing three key nodes in primary metabolism to shift metabolic fluxes toward enhanced rapamycin production: fatty acid (FA) synthesis, tricarboxylic acid (TCA) cycle, and aromatic amino acid (AAA), which are competing with the rapamycin biosynthetic pathway over precursor molecules such as malonyl-CoA and methyl-malonyl-CoA. The EQCi-based individual repression of key genes in the TCA, FA and AAA resulted in increased rapamycin titres and had little or no effect on cell growth. Validation of the EQCi circuit was performed targeting three genes simultaneously on rapamycin production. Interestingly, the EQCi circuit harbouring the gRNA combination of three genes generated the highest titre (∼660%) of rapamycin compared to controls. Repressing the transcript levels of these target genes confirmed that they were dynamically regulated in a cell density-dependent manner.

Additionally, the applicability of the EQCi circuit was demonstrated with actinorhodin, a polyketide produced by *S. coelicolor* [78]. Remarkably, the strain carrying the same EQCi system targeting *gltA* gene (*sco2736*) from the TCA cycle showed ∼850 % more actinorhodin than the wild-type [78].

In summary, incorporating both the QS system and CRISPRi allows an adjustable, completely autonomous, and dynamic downregulation of single or multiple pathways leading to metabolic flux redirection, thus increasing target product biosynthesis without further interventions. However, off-target effects of the gRNAs may result in unforeseen changes in metabolic flux as various genes are silenced simultaneously. The result may be an insufficient supply of metabolites for primary metabolism and lead to growth retardation and lower production yields. Therefore, a way forward will be to conduct quantitative studies where various gRNAs are created for each gene and using sequencing to determine the best position for each gRNA for the desired level of expression. Such quantitative datasets could enable computational models for gRNA placement. Overall, this tool holds the potential to become a universal technology for other industrial microorganisms.

Taken together the development of both data analysis tools to mine the ever-increasing amount of available data from genomes, transcriptomes, proteomes, and metabolomes along with the much-needed advancement of synthetic biology tools and the adaptations of genetic manipulation methodologies already available in other organisms, has improved and facilitated the study of the extensive metabolic repertoire of actinomycetes. These pioneer signs of progress have a major effect on the study of SMs. The computational predictive tools and the experimental methods are intrinsically linked, as advancement in the first informs the next step of development in the second. In the following sections, we addressed the link between computational and synthetic biology approaches to understand development and regulation of specialised metabolism, and their ecological interactions.

## Specialised metabolites

Actinomycetes have evolved a complex chemical language by synthesising diverse SMs detectable by the presence of a high number of BGCs in their genomes [5]. For bioprospecting novel SMs, considerable resources have been invested in pursuing unique reservoirs and unveiling their striking features. For instance, rare actinomycete, which represent less frequently isolated genera than *Streptomyces* [79], have become established sources for structurally-diverse and chemically-unique SMs [80]. Special attention has also been placed into marine actinomycetes, where highly-bioactive novel taxa have been described, such as *Salinispora* spp. [81, 82]. Furthermore, underexploited environments that drive unique evolutionary pressures have also been recently investigated, such as polar regions [83], caves [84–86], and deserts [87–89].

Combining omics technology, such as genome mining and untargeted metabolomics, offer powerful means to gain insights into the natural role of SMs. One of the selected studies uses high-throughput comparative genomics of the rare genus *Nocardia* to reveal how variations on BGCs reflected differences in the chemical structure of the compounds [90]. We also discuss a second study where the chemical potential of rare actinomycetes from unusual habitats such as polar regions is explored, linking this chemical potential to their genomic information through the novel tool NPLinker [91].

The availability of big data has encouraged the design of new mining tools for uncommon BGCs classes enabling novel insights into SMs. This is the case for our selected paper that describes one novel approach based on phylogenetic relationships and evolutionary divergence within a gene cluster to guide the subsequent experimental rationale [92]. Moreover, the chemical-reverse approach can also be informative, which takes compound elucidation as the starting point to go backwards and attempt to link its production to the responsible BGC [93]. Altogether, these different approaches increase the likelihood of discovering new BGCs to access the chemical uniqueness of their respective products, both crucial to discovering novel SMs.

### Comparative genomics and metabolomics in the genus *Nocardia* [90]

A valuable approach to studying rare actinomycetes relies on performing a deeper investigation of a promising genus through high-throughput comparative genomics and metabolomics of closely related species. Männle *et al.* applied genomic tools to predict molecules produced by BGCs and compare the findings with metabolomics data to identify chemical divergences in the structure of the compounds related to variations in the BGCs. Although still a major challenge, authors merged several data sets obtained from different technics and tools, such as antiSMASH, BiG-SCAPE, LC-MS/MS, GNPS, and NMR, by studying a conserved metabolic pathway from *Nocardia* strains.

First, phylogenetic inferences of over 100 *Nocardia* genomes from diverse sources were constructed based on conserved housekeeping genes, retrieving six major clades. Correlation between BGCs abundance and the respective clades was observed, and BGCs diversity was further investigated by applying BiG-SCAPE. As several GCFs were widespread in *Nocardia* genomes, authors focused on a conserved cluster predicted to produce nocobactin-like siderophores. Strict adjustment of BiG-SCAPE threshold similarity parameters resulted in the formation of gene clusters subfamilies. Thus, it was possible to observe that each subfamily of clusters had divergence in their metabolic pathway genes. Compounds biosynthesized by the BGCs of these subfamilies were predicted, and 12 *Nocardia* strains were cultivated to analyse their metabolomics profile through LC-MS/MS.

Furthermore, a molecular network was constructed using the GNPS platform, and correlated with the GCFs network. Analysis of detected ions led to the identification of nocobactin-like compounds based on fragmentation patterns and NMR data. These experimental results supported the bioinformatic predictions, allowing the assignment of BGC candidates to derivatives of known compounds. Also, a *Nocardia* strain was identified that might produce previously undescribed nocobactin variants.

Although the authors successfully correlated the prediction and identification of various nocobactin-type compounds, they could not detect these molecules from two *Nocardia* species that bear the biosynthetic capacity (i.e*. N. araoensis* NBRC 100135 and *N. takedensis* NBRC 100417). Instead, they discovered that these strains produce mycobactins, which leaves room for further research. This study is a promising example of developing the genomic-driven discovery of new compounds in conserved biosynthetic pathways. It demonstrates that through computational predictions and wet-lab experiments, BGCs investigation by similarity networks can predict structural variations in SMs.

### Comparative metabologenomics analysis of polar Actinomycetes [91]

Although computational networking approaches such as GNPS and BiG-SCAPE are widely used tools for large-scale metabolomics and BGCs data analyses, respectively, the importance of linking these datasets is just beginning to be recognized [94]. In this sense, Soldatou *et al.* applied the novel platform NPLinker [95], an unsupervised method for integrating paired omics data, to establish links between complex metabolomics and genomics datasets relating to marine polar actinomycetes [91].

In a previous report, authors investigated the diversity of strains in marine sediments from the Arctic and Antarctic using culturable and metagenomics approaches, which leads to a collection of 50 actinomycete strains [83]. Soldatou *et al.* went a step further into exploring the chemical potential of twenty-five strains of these phylogenetically and geographically diverse polar rare actinomycetes. In an attempt to discover chemical diversity, underexplored genera -in comparison to *Streptomyces* [79]- were selected, including *Pseudonocardia* spp., *Micrococcus* spp., *Rhodococcus* spp., and *Microbacterium* spp. By applying the one strain many compounds approach (OSMAC), a collection of 100 extracts was produced and analysed via LC-MS/MS. Then, the distribution of parent ions as an equivalent of detected metabolites was correlated to bioactivity against ESKAPE pathogens. However, it was found that bioactivity was not necessarily related to the highest number of parent ions. A molecular networking analysis, which included the MolNetEnhancer workflow [96], was carried out to assess the chemical diversity and cluster the paired ions into MFs.

Additionally, genome mining for 17 strains sequenced by Illumina technology revealed a total of 133 BGCs, where 67 % showed none or low homology to the MIBiG repository [97], suggesting novelty on the biosynthetic potential of the analysed strains. After clustering the BGCs into GCFs using BiG-SCAPE, NPLinker was used to suggest potential links between GCFs and MFs based on two standardised scoring functions. This approach made it possible to predict four metabolites, including the known compounds ectoine and chloramphenicol, whose experimental validation awaits to be confirmed.

Soldatou *et al.* show how the study of phylogenetically diverse polar actinomycetes can lead to the discovery of novel chemistry. For instance, marine *Pseudonocardia* and *Rhodococcus* strains were identified as potential sources of bioactive metabolites for further studies. Despite their interesting bioactivity profile, *Pseudonocardia* strains were excluded from the genomic analysis due to difficulties in assembling a complete genome from short reads, which would be interesting to cover in future.

Although one of the major limitations of the metabologenomic approach was the relatively low number of publicly available experimental datasets, the proposed methodology proved to be an effective tool for strain prioritisation, thus constituting a promising strategy for accelerating the discovery of novel chemistry derived SMs. Undoubtedly, further integration of bioactivity with the paired omics data will strengthen the possibility of identifying strains capable of producing new chemistry in future culture collection screenings.

### Evolution-guided discovery of antibiotics that inhibit peptidoglycan remodelling [92]

Big data analyses can be overwhelming, and thus prioritisation of BGCs to guide SMs discovery remains a continuous challenge. One strategy to accomplish BGCs selection is phylogeny-guided, as biosynthetic genes that diverge during evolution may produce compounds with new biological activity. An interesting example was demonstrated for glycopeptide family (GPAs) antibiotics, where phylogenetic reconciliation allowed us understand their origin and evolution [98]. Culp *et al.* investigate GPAs evolutionary dynamics by constructing a phylogenetic analysis of all shared genes from BGCs of GPAs and highlighting the evolutionary divergences observed in NRPS condensation domains [92]. Data were correlated with the presence/absence of self-resistance genes, which unveiled that BGCs with resistance determinants are commonly present in true GPAs and grouped into a single clade. Interestingly, two additional clades lacking known GPA resistance genes were further studied: while one was found to be related to a known compound called complestatin; the other had no associated molecule, leading to the isolation and identification of a new compound, carbomycin.

Both compounds, complestatin and carbomycin, were experimentally demonstrated to be members of the GPA family active against Gram-positive bacteria, including the vancomycin-resistant *Enterococcus* sp. and other drug-resistant clinical strains. Moreover, the mechanism of action of these compounds was described for the first time, showing they bind to peptidoglycan and block the autolysins enzyme action that is essential for cell wall remodelling. These enzymes are responsible for peptidoglycan cleavage during cell division and have a role in cell wall turnover [99]. Hence, blocking these enzymes results in bacterial growth inhibition. In addition, despite attempts to select resistant mutants to both complestatin and carbomycin compounds, resistance evolved slowly on the tested bacteria. Finally, topical lotions containing both compounds were developed and tested *in vivo* against superficial infections in mice skin, where it also was found to be effective in reducing bacterial growth.

Overall, this study demonstrates the potential of analysing BGCs evolutionary divergences for aiding and guiding cluster prioritisation. When combined with the analysis of resistance determinants of a specific antibiotic class, it can lead to discovering new compounds and novel mechanisms of action, contributing to the advancement in the treatment of drug-resistant infections. Nevertheless, dealing with a novel mode of action needs further investigation in the steps that are being blocked at the molecular level, making the investigation even more challenging. However, this study stands as a light of hope to face the rediscovery problem, not only due to the delivery of a novel antibiotic but also, elucidating modes of action.

### A biaryl-linked tripeptide from *Planomonospora* reveals a widespread class of minimal RiPP gene clusters [93]

RiPPs have drawn attention due to their intricate three-dimensional structures and broad distribution across actinomycetes [20]. RIPPs BGCs encompass a gene encoding the precursor peptide and genes encoding enzymes that modify that peptide. The precursor peptide is ribosomally synthetised, modified by enzymes included in the corresponding RIPP BGCs and activated by cleavage of the N-terminal leader peptide from the C-terminal core peptide [100]. Zdouc *et al.* discovered the smallest coding gene described to date, belonging to a new class of RiPPs minimal BGCS which remained undetected using well-established software pipelines.

Stemming from metabolomic studies of *Planomonospora* strains from Naicons’ strain collection [101], Zdouc *et al.* purified and elucidated the structure of a novel tripeptide with an unusual carbon-carbon (C-C) bond between amino acids. The authors propose to call this novel class of compounds biarylitides, since the products feature a C-C bridge between the aromatic amino acid residues. The gene cluster responsible for such a product could not be identified using the antiSMASH platform, so a tailored approach was needed to unveil the region coding for enzymes that produce biarylitides. Heterologous expression proved that a minimal gene cluster consisting of only two genes is sufficient to the develop the mature product in a heterologous *Streptomyces* host. Biosynthesis was described as follows: first, the pentapeptide precursor is ribosomally synthesized from the smallest gene ever to be described, *bytA*, accounting for solely 18-base pairs. Then, the C-C bond between the aromatic moieties of the third and fifth amino acid is carried out by *bytO*, coding for a cytochrome P450 monooxygenase. The cleavage of the two leading amino acids and N-acetylation of the mature product are likely to be carried out by other enzymes from central metabolism.

Surprisingly, bioinformatic analysis of 3,300 genome sequences revealed that the class of minimal RiPP clusters described by Zdouc *et al.* are widely distributed across various actinomycete genera. However, the biological role of this BGC and the product remains to be understood, as well as its applicability. Investigating the mechanism and specificity of the biaryl crosslink might have important uses in stabilizing peptides for different applications. This study highlights the importance of metabolomics-guided studies in discovering novel BGC types, without the need for including bioactivity assays in the metabolite prioritisation step to purify and describe an interesting compound. Undoubtedly, this study highlights the usefulness of additional approaches for BGCs discovery alongside traditional genome mining tools, which simply cannot predict unprecedented BGCs. Furthermore, this opens the road for their future evolutionary and comparative genomic study.

Overall, these studies stand as solid examples of how integrating genomics and metabolomics data analyses can provide unique insights that otherwise remain hidden when datasets are analysed independently. Regarding rare actinomycetes, unique environments or not-so-common classes of compounds like RiPPs or GPAs, are examples of how unconventional approaches may unveil new opportunities for discover novel compounds. Moreover, the study of SMs needs to be considered as a whole, understanding that while they might be important in the clinical setting, their primordial role probably relies on a fundamental metabolic function within bacteria, which can be revealed through the understanding of actinomycete’s complex development, which will be discussed in the next section.

## Development and regulation

Unlike other bacteria, filamentous actinomycetes such as *Streptomyces,* possess a strikingly complex life cycle. Briefly, a germinating spore establishes a multicellular network of vegetative mycelium by growing through hyphal tip extension and branching. After depletion of nutrients, it undergoes autolytic degradation forming aerial hyphae followed by segmentation into spore chains that will spread to start a new developmental cycle [3]. Phenotypic differentiation also leads to genetic heterogeneity due to the high-frequency of rearrangements and deletions [102]. Although this phenomenon was known a time ago, one of the studies selected for this review linked this phenotypic differentiation to a division of labour within *Streptomyces* colonies, giving rise to enhanced capacity of antibiotic production [103].

SMs production is also correlated with *Streptomyces* morphogenesis at the onset of sporulation, being another evidence of how stress and developmental programmes are intimately connected [104]. Master regulators, such as Bld (aerial hyphae formation) and Whi (spore formation) are amongst the molecular mechanisms governing development, involving complex regulatory cascades [105–107]. Additionally, nucleotide second messengers, such as p(p)ppGpp, cAMP, c-di-AMP and bis-(3´,5´)-cyclic di-guanosine monophosphate (c-di-GMP), play a major role in coordinating the transition between vegetative filamentous hyphae growth, reproductive aerial mycelium, and subsequent differentiation into spore chains [108]. Recently, transcriptional profiles of hyper-sporulating and delayed-development mutants revealed novel key genes responsive to c-di-GMP dynamics [109], discussed in another article we selected. Ultimately, to understand the complex regulation of the life cycle of actinomycetes is paramount to take advantage of the full potential of these antibiotic-producers’ organisms. Moreover, this may be considerably improved with a better understanding of the basic underlying regulatory mechanisms, an area where further study is needed.

### Antibiotic production in *Streptomyces* is organized by a division of labour through terminal genomic differentiation [103]

Although the phenomenon of genomic instability was discovered in *Streptomyces* decades ago [110], it has remained poorly understood. Zhang *et al.* describe how this genetic diversification is intertwined with a division of labour in *Streptomyces* colonies, causing irreversible differentiation that gives rise to mutants with enhanced antibiotic production capabilities.

In colonies starting from a single spore, up to 2 % of cells, depending on media and growth conditions, undergo vast genetic rearrangements found on both chromosomal ends that lead to an increase in antibiotic production. Genome rearrangements can vary in size and frequency, while deletions can cause a loss of up to 1,000 genes; amplification of regions also occurs, containing various gene copies flanked by an insertion sequence (i.e*.* IS1649, encoded by SCO0091 and SCO0368). While each mutant has a unique profile of inhibition against other soil-dwelling competitors, their ability to form spores becomes greatly hampered. Moreover, through these specialised mutants, the parent strain gains an advantage by maximising both the colony-wide antibiotic production and its diversity.

The link between genomic instability and phenotypic heterogeneity was established by sequencing morphologically distinct daughter colonies of *S. coelicolor* M145. Instead of reverting their morphology back to wild-type, as would be expected in the case of a clonal population, the progeny of aberrant colonies was hypervariable. Sequencing revealed that mutants contained profound chromosomal changes, involving insertions and deletions, from both arms of the chromosome. In addition, the authors established an elegant method for deducing the minimal deletion size from phenotype, by linking arginine auxotrophy and chloramphenicol susceptibility, by using pulse field gel electrophoresis (PFGE). Furthermore, pigmented antibiotics from *S. coelicolor* M145 were used as indicators of bioactivity, confirmed by overlay assays against *Bacillus subtilis *. Additional experiments of mixing spores from wild-type and mutant colonies in known proportions showed a strong positive correlation between the percentage of mutants in the starting population and the inhibition zone size. When measuring the overall spore count of such mixed colonies, no significant reduction was observed until mutants comprised up to 50 % of the starting population, thereby neutralising the high individual fitness cost for the colony. Interestingly, the final frequency of mutant spores declined to less than 1%, even if the starting proportion was as high as 80 %. In the future, it will be interesting to see if this phenomenon is also true for non-pigmented antibiotics.

The work of Zhang *et al.* shows that *Streptomyces* colonies starting from a single spore become genetically diverse with daughter cells specialising in specific tasks, thus providing for the first-time evidence of division of labour in antibiotic production and spore formation. The molecular mechanisms regulating these rearrangements remain to be discovered. Furthermore, it would be relevant to understand the cues triggering these rearrangements for the manipulation of antibiotic producing strains, as they might depend on external signals and/or be context dependant. We are looking forward to follow-up studies as preliminary results on *Streptomyces* environmental isolates hint towards a widespread nature of such division of labour.

### Specialized and shared functions of diguanylate cyclases and phosphodiesterases in *Streptomyces* development [109]

In *Streptomyces *, c-di-GMP is a key life cycle regulatory factor [111] that coordinates cell-fate via a complex signalling network: while BldD-(c-di-GMP) acts as a transcriptional regulator of vegetative filamentous growth [112]; initiation of sporulation is determined by the activity of the σ^WhiG^ RsiG-(c-di-GMP) complex [113]. Turnover of the second messenger c-di-GMP is mediated by two sets of enzymes with specific catalytic domains [114]: diguanylate cyclases (DGCs) that catalyse its synthesis, and phosphodiesterases (PDEs) that are involved in its degradation [115].

Although c-di-GMP-sensors are known, there is a gap in understanding which genes are responsive to c-di-GMP dynamics. Haist *et al.* aimed to unveil the specific molecular targets in two DGCs mutants (Δ*cdgB* and Δ*cdgC*) driving hyper-sporulation, and two PDEs mutants (Δ*rmdA* and Δ*rmdB*) delaying development [114]. RNA-seq was used to compare mutants' transcriptional profiles with wild-type *S. venezuelae *, and further biochemical experiments were developed to confirm the function of the differentially expressed genes.

Results demonstrate that c-di-GMP has a global regulatory role, antagonistically controlled by the DGCs (CdgB and CdgC) and the PDEs (RmdA and RmdB). Despite observing shared enzymatic activities between the two DGCs and the two PDEs, respectively; each one controls a characteristic set of genes, representing unique roles in developmental regulation and thus explaining their differential phenotypes. Unexpectedly, few *bld* and *whi* genes, known to be key regulators of the developmental regulatory cascade, were differentially expressed in the mutant strains. On the contrary, two other categories were significantly affected: the expression of the hydrophobic sheath, controlled via chaplin (*chp*) and rodlin (*rdl*) genes; and the cell division components, through *ftsZ* expression.

Overall, Haist *et al.* demonstrate how PDEs (RmdA and RmdB), and DGCs (CdgB and CdgC) differentially govern development via transcriptional BldD and σ^WhiG^ fine-tuned sensing of c-di-GMP, contributing to our understanding of the c-di-GMP multilayered cascade. In addition, expression of c-di-GMP-responsive genes revealed insights into how they are involved in the coordinated progression of *Streptomyces’* life cycle. While cell division genes control the specific timing of when spores are formed, such as *ftsZ*; the determination of the sporulation mode, either being in the aerial hyphae or out of substrate mycelium, is induced through chaplin and rodlin genes, which are involved in the formation of the hydrophobic sheath that cover aerial hyphae and thus control where spores are developed.

Furthermore, a link between c-di-GMP signals and antibiotic genes expression was proposed, by studying of the chloramphenicol pathway. Chloramphenicol BGC involves 17 *cml* genes [116], which were significantly downregulated in the two mutants driving hyper-sporulation through the repression of *bldM*. However, *cml* genes were not observed affected in the two delayed-development mutants. Recently, the modulation of c-di-GMP has demonstrated to be an appealing candidate to manipulate antibiotic production [117]. Thus, expanding how c-di-GMP affects other antibiotic pathways, along with the effect on c-di-GMP-responsive genes remains for further investigation.

Understanding the complex regulatory networks that underlie the developmental life cycle is crucial to uncover the full potential of these producer organisms. These studies show that it is fundamental to gain knowledge into the molecular effectors that can trigger SMs synthesis. These insights can subsequently inform metabolic engineering strategies for improved antibiotic production. Furthermore, in addition to the internal regulation of SMs synthesis, biotic and abiotic environmental cues can also trigger the activation of certain BGCs, leading us into the final section of this review where we focus on ecology and host interactions. Deciphering actinomycete interactions either with other micro- and/or macro-organisms will increase the understanding on how SMs act as signalling compounds. Moreover, a better knowledge of interspecies interactions will further unlock the huge chemical diversity of actinomycete SMs. Altogether, it is becoming clear that a deep understanding of all intra- and extra-cellular factors affecting BGCs expression and SMs biosynthesis are necessary to further our comprehension into actinomycete research.

## Ecology and host interactions

Although actinomycetes are well-known as antibiotic producers, most BGCs remain silent in controlled conditions [38]. There is little ecological rationale for the constitutive production of these energetically expensive metabolites when grown in favourable conditions such as high nutrient media. Moreover, SMs production is generally under strict regulatory control, meaning that several cues triggering BGCs expression can be absent or even repressed [118, 119]. However, in nature, actinomycetes are part of complex microbial communities and globally there is a lack of understanding of these community dynamics. Additionally, actinomycetes are ubiquitous in several habitats, facing ecological adaptations that affect genetic determinants expression. Identifying ecologically relevant interactions is further complicated when symbiosis with macro-organisms such as plants, fungi and animals is considered. Overall, these interactions control the expression of actinomycete BGCs, which in turn, have played a major role in the evolution of the vast chemical diversity of SMs [120]. Co-culture experiments attempt to mimic these complex relationships in the laboratory and thus elicit the production of SMs [11]. Therefore, we selected one article where the co-culture approach of *Salinispora tropica* with phytoplankton led to the decryption of two BGCs [121].

In soil, *Streptomyces* species are free-living bacteria that play an important role in decomposing of organic matter by breaking down complex materials [10]. Geosmin is a widespread metabolite known long ago as the responsible for conferring *Streptomyces* their earthy odour. However, until now, the ecological role of geosmin was cryptic. A highlight from 2020 was that first insights into geosmin role in nature were uncovered, by an article we selected that demonstrate how geosmin production is closely linked to *Streptomyces* developmental cycle, where is shown that geosmin is used to attract springtails that help to disperse bacterial spores over longer distances [122].

Additionally, actinomycetes have been recognized as defensive symbionts of several invertebrate species [123]. They have been shown to interact with plant roots, where they have evolved beneficial interaction for protection against phytopathogens [120]. For example, *Streptomyces* can enter the root tissue and enable plants to compete for food by enhancing root exudates [10]. Moreover, they can exert plant growth-promoting (PGP) activities, by increasing plant biomass through direct or indirect manners, such as phosphate mobilization, nitrogen fixation, iron acquisition, or enhancing the production of plant hormones, to name just a few [124]. The study we have selected highlights the potential of *Streptomyces* as plant probiotics in *Arabidopsis thaliana*, which can further be applied to commercial crops [125]. Altogether, these important interactions reflect the natural purpose of harbouring a large repertoire of chemical diversity and how these SMs have evolved as a direct product of these interactions.

### Phytoplankton trigger the production of cryptic metabolites in the marine actinobacterium *Salinispora tropica* [121]

Antagonistic interactions between the marine-obligate *Salinispora tropica* and co-occurring heterotroph bacteria have been previously reported [126]. The induction of antibiotic activities has been linked to temporal changes in compound production composition through a complex pattern that varies regarding the interaction and type of metabolites [127]. Chhun *et al.* extend this co-culturing approach by adding insights into inter-species interactions between *S. tropica* and marine phototrophs. Through the integration of co-culture, metabolomics and proteomic techniques, phototrophs' effect on *Salinispora* BGCs expression is investigated.

Chhun *et al.* reported that *S. tropica* CNB-440 affects the growth of a taxonomically diverse group of phytoplankton which includes the model species *Synechococcus* sp. WH7803 (cyanobacteria); *Emiliania huxleyi* (coccolithophore); and *Phaeodactylum tricornutum* (diatom). A co-culture experiment using a porous membrane to separate the organisms physically suggested that secreted metabolites of *S. tropica* importantly affected *Synechococcus* sp. proliferation. Furthermore, metabolomic analyses of co-cultures of *S. tropica* and *Synechococcus* sp. revealed the production of eight metabolites that were not detected in axenic culture: four were related to the known compound salinosporamide, whilst the other four were identified as potentially novel. As an attempt to identify BGCs overexpressed in the phototroph co-culture, a comparative proteomic analysis was carried out with the proteome of *S. tropica* exposed to phytoplankton photosynthate (i.e*. Synechococcus* culture supernatant, rich in phototroph-released nutrients). Besides confirming salinosporamide’s BGC (*sal*) overexpression, several proteins encoded in orphan BGCs were detected in the photosynthate culture. A polyketide synthase (*pks3*) and a non-ribosomal peptide synthetase (*nrps1*) cluster were identified as putatively responsible for metabolite biosynthesis.

Chhun *et al.* illustrate the importance of an integrative approach in the study of actinomycete ecology. It was demonstrated that metabolites produced by the rare actinomyces marine *S. tropica* CNB-440 have the potential to kill both prokaryote and eukaryote phytoplankton. Although the experimental model may not represent exactly the ecological interactions found in the ocean, the photosynthate cultures are a good approximation to test the effect of phytoplankton over the expression of BGCs in *Salinispora *. Moreover, it was shown that co-culturing phototrophs and marine-derived actinomycetes could accelerate the discovery of novel bioactive metabolites. Metabolomics analysis was used to identify the overexpressed metabolites in co-cultures, allowing the identification of four putative candidates. Metabolites potentially encoded in *nrps1* and elicited by photosynthate showed promising antimicrobial activity. Unfortunately, it was not possible to link a particular overexpressed metabolite with this BGC, thus awaiting further identification. Besides expanding the knowledge on the specialised metabolites produced by *Salinispora *, this study provides new insights into the ecology of actinomycetes in marine environments.

### Developmentally regulated volatiles geosmin and 2-methylisoborneol attract a soil arthropod to *Streptomyces* bacteria promoting spore dispersal [122]

Geosmin is a sesquiterpenoid metabolite known for giving soil its characteristic earthy-smelling odour, produced by several actinomycetes [128], amongst other microorganisms. Its biosynthesis involves a geosmin synthase, which catalyses the cyclization of farnesyl diphosphate to germacrene D and germacradienol, and then converts the latter to geosmin [129]. Interestingly, genes for its biosynthesis are highly conserved in all *Streptomyces* genomes [130]. Similarly, genes for the biosynthesis of another earthly-smell molecule, the monoterpene 2-methylisoborneol (2-MIB), are found in approximately half of sequenced *Streptomyces* genomes [130]. The ubiquity of producing these volatile organic compounds (VOC) in streptomycetes suggests that they confer a selective advantage. However, the benefit for the producer bacteria and, therefore, geosmin’s biological role remained unknown.

Becher *et al.* investigated the role of VOCs in the context of *Streptomyces* interaction with soil-dwelling arthropods, the springtails. In field experiments, springtails showed a significant attraction to traps baited with *Streptomyces coelicolor* colonies, further confirmed in laboratory Y-tube bioassay. Chemosensory responses in springtail antennae gas chromatography (GC) combined with electrophysiological antennal detection (EAD) revealed that geosmin, germacradienol, germacrene D, and 2-MIB induce sensory responses. Mutants of these VOCs (Δ*geoA*; Δ*mibAB*; and Δ*geoA* Δ*mibAB* double mutant) demonstrated that both earthy odorants are behaviourally active and serve as attractants for the springtail, guiding them for localization of food sources.

Transcriptome analyses unveil that these VOCs are intimately connected to *Streptomyces* developmental life cycle. Expression of *geoA* and *mibA-mibB* depends on the regulatory gene *bldM*, involved in aerial hyphae and spore’s development [131], further confirmed by lack of production of geosmin and 2-MIB in a Δ*bldM* mutant. Interestingly, *mibA-mibB* genes were found to form an operon with *eshA*, encoding for a cyclic nucleotide-binding protein of unclear function, which remains to be characterised. ChIP-seq showed that BldM directly regulates the *eshA-mibA-mibB* promoter; however, it does not regulate *geoA* which instead is directly regulated by WhiH, involved in correct septation of aerial hyphae during spore formation [132].The production of both earthy odorants is directly coupled with spore formation via transcriptional control of key sporulation regulators.

The geosmin- and 2-MIB-mediated attraction of springtails and the correlation of these VOCs to spore formation suggested that springtails might act as vectors for spore dispersal. Authors confirmed this observation by demonstrating that it occurs via two different routes: adherence to their body surface cuticle; and passage through the gastrointestinal tract and defecation. Therefore, *Streptomyces* benefit from emitting geosmin and 2-MIB as part of their developmental programme as these volatile scents guide springtails to sporulating microcolonies, where they serve as vectors for spore dispersal over long distances. It remains to be discovered if this relation can be also observed in another soil insects or even more, expanded to other co-inhabiting animals. Further research is needed to test *Streptomyces *-springtails interaction in a community context, and to explore the role of other VOCs may act as either attractants or repellents for a comprehensive understanding of chemical ecological complex interactions.

### *Streptomyces* endophytes promote host health and enhance growth across plant species [125]

*Streptomyces* have been previously reported to interact with plant roots either through the rhizosphere, which is the root surrounding soil; or the endophytic compartment, the niche within and between root cells [124, 133]. Recent studies in *Arabidopsis thaliana* have revealed that streptomycetes are present and enriched in the endophytic compartment relative to that in the bulk soil [134, 135]. Streptomycetes have also been isolated from the endophytic compartment of numerous other plant species, including crops. Taking together, both the ability of *Streptomyces* to colonize plant roots along with the extensive repertoire of SMs, makes them interesting candidates for biocontrol in crops, with a possible application as plant probiotics. To this end, Worsley *et al.* isolated *Streptomyces* endophytes from *A. thaliana* roots to test the hypothesis of beneficial effects on the host and test these strains on commercial crops plants, such as wheat.

Five strains were isolated from *A. thaliana* root microbiome (i.e*.* L2, N1, N2, M2 and M3) and compared to another three strains of the known endophyte *Streptomyces lydicus *, all selected for genome sequencing. First, eGFP-tagged *Streptomyces* strains were re-infected into *A. thaliana* roots to verify the ability of the endophyte to recolonise plants. Then, all the genome-sequenced strains were inoculated into seedlings to investigate their effect on growth. It was observed that strains were able to colonize the root surface, having a significant effect on plant biomass: strains L2, M2, and M3 gave the best enhancement of dry biomass, while the three *S. lydicus* strains had no effect. Moreover, strains N1 and N2 decreased *A. thaliana* growth. Interestingly, plant-treated with a mixture of L2, M2, and M3 gave higher growth in comparison with individual and uninoculated controls, respectively.

Genome mining and bioassays of all the eight sequenced strains revealed that they produce PGP compounds, including indole-3-acetic acid (IAA) and ACC deaminase. Moreover, they possess several BGCs involved in siderophores and antimicrobial compounds. Among them, strain N2 shows a broad-spectrum antimicrobial activity and produces filipin-like polyenes, including 14-hydroxyisochainin. N2 antifungal activity was improved two-fold in the presence of IAA, suggesting that N2 may display its activity in proximity to the plant root when competing with other microbes. Additionally, coating wheat seed with N2 protects the germinating seedlings from the take-all fungus *Gaeumannomyces graminis var*. *tritici*, an economically important wheat pathogen.

In summary, this study demonstrated that introducing certain *Streptomyces* into the root microbiome provides significant benefits to host plants. However, two of the five root-associated *Streptomyces* strains showed a negative *in vitro* effect regarding *A. thaliana*’s biomass. Therefore, the effects of *Streptomyces* strains on host plants seem to be strain-dependent, and further research is needed to understand their role in these interactions. Nevertheless, the authors achieve to develop a system for studying the role of SMs in nature that may provide new insights to activate silent BGCs. Specifically, they demonstrate that IAA increased the antifungal activity of one of the root-associated *Streptomyces* strains, providing evidence of how external signals can affect the expression of SMs. In addition, exploring plant-* Streptomyces* interactions led to the identification of novel PGP and biocontrol agents that could be developed as active biofertilizers, as greener alternatives to pesticides currently having a negative impact in ecosystems. In future, it will be interesting to expand the testing and explore the effects of *Streptomyces* in other crops.

## Conclusion

This review highlights recent research that uses integration of perspectives to further understand actinomycete biology, focusing on four broad areas: *i)* technology and methodology; *ii)* specialised metabolites; *iii)* development and regulation; and *iv)* ecology and host interactions.

In the last decade, the genomic era opened a new roadmap for the study of SMs in actinomycetes. The accessible cost of whole-genome sequencing enabled the exploration of not only few, but whole collections of strains. Over a relatively short period of time, the amount of available genomic data increased exponentially and is bound to continue. Likewise, other omics technologies such as metabolomics and transcriptomics have experienced a similar data expansion. Consequently, it is no surprise that efforts are focused on developing computational tools to handle this big data. BiG-SLiCE and BiG-FAM have recently expanded the BiG-SCAPE family, enabling us to compare millions of BGCs, and group them into gene cluster families. Moreover, additional tools such as Qemistree, which allows hierarchical tree clustering based on chemical similarity; and NPLinker, that link both genomic and metabolomic data, permits actinomycete research to approach old problems from a different angle, such as the rediscovery of known compounds as well as prioritisation of novel BGCs.

In parallel to computational advances, synthetic biology tools such as a *Streptomyces* tailored cell-free toolkit; and the combination of CRISPR technology with quorum sensing as a means for metabolic engineering, are crucial to test the prediction-based hypotheses using wet-lab experiments. These approaches will continue to increase our understanding of the role of newly characterised BGCs. This information can then be stored in a database such as MIBiG, which will increase our ability to conduct robust comparative studies. Additionally, synthetic biology will be a key to access silent or cryptic BGCs as well as those that are found in unculturable organisms by providing controlled conditions.

The importance of applying these latest cutting-edge technologies to fundamental actinomycete research questions has already been revealed through various major findings. This review covering just over a year of research in this field offers plenty of examples such as genomic differentiation as means to achieve division of labour for antibiotic production in *Streptomyces* colonies; a deeper understanding of the c-di-GMP regulatory cascade and the role of the enzymes involved; the use of metabologenomics focussing on a diverse phylogenetic group from polar regions and in the rare genus *Nocardia *, leading to the discovery of new compounds; or using evolutionary divergence in gene clusters for prioritisation of interesting BGCs, as well as the reverse approach starting from the structure of a novel metabolite to find the gene cluster responsible for its production. Finally, we also highlight the impact on ecological research, particularly how co-culturing can lead to the production of previously silent metabolites; how *Streptomyces* can boost plant health; and discovering the environmental role of the widely distributed geosmin.

The emergence of the SARS-CoV2 global pandemic has recently put microbiology research at the heart of finding solutions for global challenges. Microbiology research will play a key role not only during the current difficult times but also in tackling future pandemics and alleviating the devastating effects that climate crisis will bring. Another major emergency in the making is the ever-increasing levels of drug resistance. Thus, our community needs to rationally exploit the potential for discovering new compounds harboured by our beloved actinomycetes and unveiling their many other secrets. Our unprecedented times reflect the need for us to act as one community, collaborate by interconnecting and overlapping knowledge from different research fields, and ultimately integrate them into shared solutions for common problems. We hope that you can see the parallels on how integrating perspectives in the actinomycete field has led to new discoveries and that there is a motivation for this to continue as a driving force in the future of actinomycete research and more widely, within microbiology.
